# Exposure to the complement C5b-9 complex sensitizes 661W photoreceptor cells to both apoptosis and necroptosis

**DOI:** 10.1007/s10495-015-1091-7

**Published:** 2015-01-21

**Authors:** Hui Shi, Jennifer A. E. Williams, Li Guo, Dimitrios Stampoulis, M. Francesca Cordeiro, Stephen E. Moss

**Affiliations:** 1 Department of Cell Biology, UCL Institute of Ophthalmology, 11-43 Bath Street, London, EC1V 9EL UK; 2 Department of Visual Neuroscience, UCL Institute of Ophthalmology, 11-43 Bath Street, London, EC1V 9EL UK; 3 Department of Ophthalmology, Tongji Hospital, Tongji Medical School, Huazhong University of Science and Technology, Wuhan, China

**Keywords:** 661W, Apoptosis, Necroptosis, Complement, c5b-9, Retina

## Abstract

The loss of photoreceptors is the defining characteristic of many retinal degenerative diseases, but the mechanisms that regulate photoreceptor cell death are not fully understood. Here we have used the 661W cone photoreceptor cell line to ask whether exposure to the terminal complement complex C5b-9 induces cell death and/or modulates the sensitivity of these cells to other cellular stressors. 661W cone photoreceptors were exposed to complete normal human serum following antibody blockade of CD59. Apoptosis induction was assessed morphologically, by flow cytometry, and on western blotting by probing for cleaved PARP and activated caspase-3. Necroptosis was assessed by flow cytometry and Sirtuin 2 inhibition using 2-cyano-3-[5-(2,5-dichlorophenyl)-2-furyl]-*N*-5-quinolinylacrylamide (AGK2). The sensitivity of 661W cells to ionomycin, staurosporine, peroxide and chelerythrine was also investigated, with or without prior formation of C5b-9. 661W cells underwent apoptotic cell death following exposure to C5b-9, as judged by poly(ADP-ribose) polymerase 1 cleavage and activation of caspase-3. We also observed apoptotic cell death in response to staurosporine, but 661W cells were resistant to both ionomycin and peroxide. Interestingly, C5b-9 significantly increased 661W sensitivity to staurosporine-induced apoptosis and necroptosis. These studies show that low levels of C5b-9 on 661W cells can induce apoptosis, and that C5b-9 specifically sensitizes 661W cells to certain apoptotic and necroptotic pathways. Our observations provide new insight into the potential role of the complement system in photoreceptor loss, with implications for the molecular aetiology of retinal disease.

## Introduction

The complement system acts as the frontline defence against invading pathogens, and it facilitates the removal of modified and dead host cells upon activation by three distinct modalities, which are the classical, the alternative and the lectin pathways [1, 2]. However, individual complement proteins are increasingly linked with distinct activities that lie beyond their roles in host defence. For example, the classical pathway, through the activity of C1q, has been shown to play a key developmental role in synaptic pruning in the central nervous system (CNS) [3], and complement proteins are widely reported to be up-regulated in a variety of CNS diseases [4]. Although each pathway has its own mechanism of activation that enables it to distinguish between foreign and host cells, all converge on the formation of the terminal C5b-9 complex (also known as membrane attack complex, or MAC), which creates pores on cell membranes and in certain cell types may cause lysis and apoptosis. Formation of C5b-9 begins with the binding of C5b6 to the plasma membrane of the target cell, with subsequent recruitment of complement proteins C7 and C8, and culminating in insertion of variable numbers of complement C9 proteins to form the lytic pore.

In general, cells protect themselves from excessive complement activation through fluid-phase regulators such as complement factor H (CFH) and cell membrane molecules such as CD59 and decay accelerating factor (DAF), which prevent the formation of C5b-9 complexes [5]. The requirement for protection against complement activation clearly exists in the retina since rod and cone photoreceptors in human eyes, and astroglial cells in mouse eyes have been shown to express DAF [6, 7], and we also reported that CD59a and CFH are up-regulated in the mouse neuroretina in normal ageing, again supporting the idea that complement regulation is required for maintenance of a healthy retina [8].

Understanding how photoreceptors die in retinal degeneration is important for the future development of therapeutics, but the mechanisms of cell death have yet to be fully elucidated. There is compelling evidence that apoptosis plays an important role [9–11], but necrotic cell death also occurs in mouse models of photoreceptor loss and other forms of neurodegeneration [10], and multiple proteases have been reported to be involved in programmed cell death in 661W cone photoreceptors [12]. Given the central importance of cone cell death in age-related macular degeneration (AMD), and the evidence of increased complement activation in AMD [13], we undertook this study to find out whether C5b-9 could induce 661W death or sensitize 661W cells to conventional apoptotic agonists. We observed that C5b-9 assembly on 661W cells led to apoptotic cell death, and that sub-lethal levels of C5b-9 increased the sensitivity of these cells to apoptosis and necroptosis induced by staurosporine, but not peroxide or ionomycin. Apoptosis and necroptosis differ in that the former refers to a series of morphological and biochemical changes, typically involving caspase activation, nuclear condensation and membrane blebbing, while the latter describes a form of unprogrammed cell death that does not involve caspases, and that is frequently associated with activation of the tissue necrosis factor-α receptor (TNFR).

## Materials and methods

### Cell culture

The photoreceptor cell line (661W) was a generous gift of the Agarwal laboratory. The line was originally provided as RGC-5 but this has since been shown to be identical to 661W [14], and this is re-confirmed here. Cells were routinely maintained in Dulbecco’s modified Eagle’s medium with glutamine (Invitrogen), supplemented with 10 % fetal bovine serum (Gibco) and 100 U/ml penicillin/streptomycin (Gibco). We undertook a PCR-based validation of the cells to exclude any possibility that they might be rat ganglion cells. The primers we used were as follows—note that all primer sequences are written 5′–3′, with the forward primer sequence first: Mouse β-globin, CCTGTGGGGAAAGGTGAAC and ATACCAGATACCTGCA GGCTTAT; Rat β-actin, GGCTTTAGGAGCTTGACAATACTG and GCATTGGTCACCTTT AGATGGA; mouse Nrl, TGTCTGTGCGCGAGTTGAAC and AAGAGGTGTCGTCTGTGTGG; mouse rhodopsin, CACTCGTTGGCT GGTCCAGGTAC and AGCCGCCTCCTTGACTGTGAAGA; mouse GNAT2, GCATCAGTGCTGAGGACAAA and CTAGGCACTCTTCGGGTGAG; mouse SWL opsin, TGTACATGGTCAACAATCGGA and ACACCATCTCCAGA ATGCAAG; mouse MWL opsin, CTCTGCTACCTCCAAGTGTGG and AAGTATAGGGTCCCCAGCAGA.

### C5b-9 formation

Seventy to eighty percentage confluent 661W cells were starved in serum-free medium for 24 h, then treated with a rabbit polyclonal anti-human CD59 antiserum (a generous gift from Prof BP Morgan, Cardiff University, Cardiff, UK) for 1 h at 37 °C. Cells were washed with PBS and incubated with normal human serum (NHS) (Jackson Immuno Research) for 1 h. Heat-inactivated (HI) NHS (30 min at 56 °C) was used as the complement-deficient control. In some experiments NHS was replaced with C7-deficient human serum (CompTech Texas, USA), in the presence or absence of complement C7 purified from human serum (Sigma). Afterwards, the cells were washed with PBS and supplied with complete medium.

### MTT viability assay

661W cells were seeded at 10^3^ cells per well in 96-well plates and serum-starved for 24 h. After the induction of C5b-9 formation (see earlier), cells were incubated with 100 µl/ml MTT (5 mg/ml thiazolyl blue) in sterile phosphate buffered saline (PBS) at 37 °C for 3 h until formazan crystals appeared. After removing the supernatant, 100 µl of DMSO (Sigma) was added to solubilize the formazan product. Absorbance was measured at 570 nm using a spectrophotometer plate reader (Fluostar Optima).

### Immunofluorescence

661W cells grown on coverslips were serum-starved as described above and induced to form C5b-9. Cells were fixed with 4 % paraformaldehyde in PBS at 4 °C for 20 min, permeabilized with 0.2 % Triton X-100 for 30 min and blocked with 1 % bovine serum albumin (BSA) (Sigma-Aldrich) at room temperature for 1 h before incubation with the anti-C5b-9 antibody (Dako, 1:50) at 4 °C overnight. The cells were then washed three times using PBS, and incubated with anti-mouse IgG secondary antibody conjugated to Alexa Fluor 488 for 1 h at room temperature in the dark. In some experiments rhodamine-phalloidin (Invitrogen, 1:200) was also applied to observe the F-actin cytoskeleton. Finally, cells were washed three times with PBS and mounted with Vectashield mounting media in the presence of DAPI (Vector Laboratories, Burlingame, CA, USA). Images were acquired using a Leica TSC-SP2 laser scanning confocal microscope (Leica Microsystems GmbH, Wetzlar, Germany).

### Western blotting

70–80 % confluent 661W cells were serum-starved in 6-well plates for 24 h, and induced for C5b-9 formation. Afterwards the cells were washed gently with PBS and incubated with 100nM staurosporine (Sigma), 1 µM ionomycin (Sigma), 4 µM chelerythrine (Sigma) or 10 µM H_2_O_2_ (Sigma) in complete medium. In some experiments the concentrations of agonists were varied as detailed in the figure legends. Cells were then lysed using RIPA buffer supplemented with protease inhibitors (Roche, West Sussex, UK). Protein concentrations were measured using the Bio-Rad Assay and 30 µg were separated by 10 % SDS-PAGE and transferred to a PVDF membrane. Antibodies against Poly (ADP-ribose) polymerase (PARP), cleaved caspase-3, GAPDH and α-tubulin were purchased from Cell Signaling Technology, Beverly, MA. Protein bands were visualized by chemiluminescence using ECL substrate (Thermo Scientific Pierce Protein Research Products).

### Flow Cytometry

For quantitation of apoptosis, cells were serum-starved in 6-well plates for 24 h and induced for C5b-9 formation. Cells were then washed gently with PBS and incubated with a range of concentrations of staurosporine or chelerythrine for 4 and 8 h. In some experiments cells were also treated with 10 µM AGK2 (Sigma). The apoptotic 661W cells were measured with an annexin V/propidium iodide (PI) apoptosis detection kit (BD Biosciences, Montreal, Quebec, Canada) according to the manufacturer’s instructions. Briefly, cells were trypsinized and pelleted by centrifugation, washed once with ice-cold PBS, resuspended in 1× Binding Buffer at a concentration of 1 × 10^6^ cells/ml, from which 100 µl of cell suspension (1 × 10^5^ cells) was transferred to a 5 ml Falcon tube. Cells were first incubated with 5 µl FITC-Annexin V and 5 µl PI for 15 min at RT (25 °C) in the dark, then 400 µl of 1× Binding Buffer was added to each tube. The cell suspension was then analysed by Cyan FACS analyser, FlowJo was used for data analysis. The results are presented as the percentage of cells that were early apoptotic (Annexin-V^+^ PI^−^) or necroptotic (Annexin-V^+^ PI^+^).

### Statistical analysis

The data are represented as mean and standard error of at least three independent experiments. Statistical comparisons were made using a two-tailed Student’s *t* test. Significant differences were defined as *P* < 0.05.

## Results

### Cell line validation

**Fig. 1:**
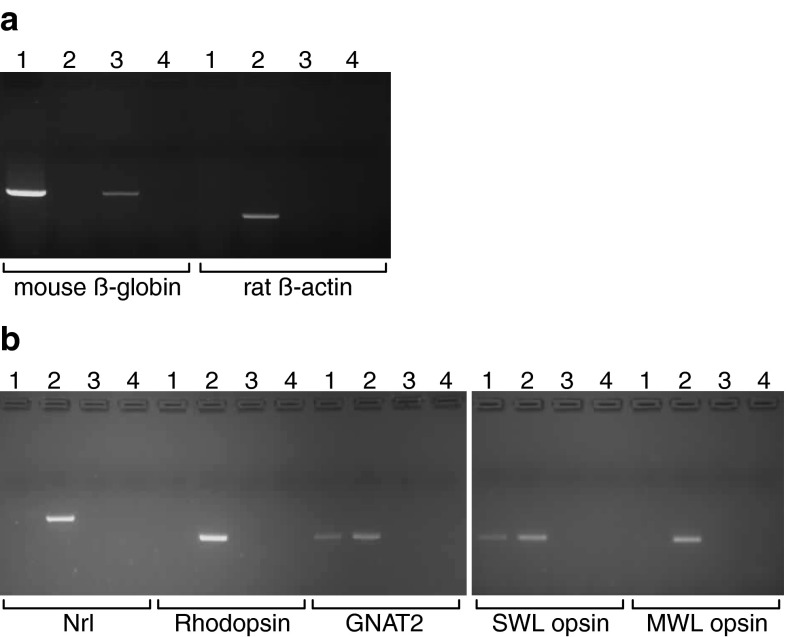
Validation of the 661W cell line. **a** We used mouse and rat genomic DNA as templates to confirm the species of the cell line, with mouse ß-globin and rat ß-actin probes respectively. *Lane 1* 661W cell genomic DNA; *Lane 2* rat genomic DNA; *Lane 3* mouse genomic DNA; *Lane 4* water (negative control). **b** We then used a set of mouse-specific retinal primers as indicated in the figure to assess expression of rod and cone markers in the cell line. *Lane 1* 661W cell cDNA; *Lane 2* mouse neuroretina cDNA (positive control); *Lane 3* mouse liver cDNA (negative control); *Lane 4* water (negative control)

Because of recent issues surrounding the identity of certain ocular cell lines [14], we used PCR to confirm that the cell line used in this study was indeed 661W. First we established that the line was of mouse origin, by using primers specific for mouse ß-globin and rat ß-actin against a genomic DNA template. Bands of the expected size were produced from our 661W cells and control mouse DNA, whereas the rat probes failed to generate a product from our cells (Fig. 1a). We then used a number of photoreceptor-specific probes, with mouse retina cDNA as a positive control in each case, to test for expression of rod and/or cone markers. Our 661W cells failed to yield products for Nrl and rhodopsin, but we observed products for GNAT2 and SWL opsin (Fig. 1b). We did not observe a PCR product using the MWL opsin probes. Taken together, these results show that our cell line is of mouse origin and that it expresses cone photoreceptor markers, consistent with it being the 661W line.

### C5b-9 formation on 661W cells

**Fig. 2:**
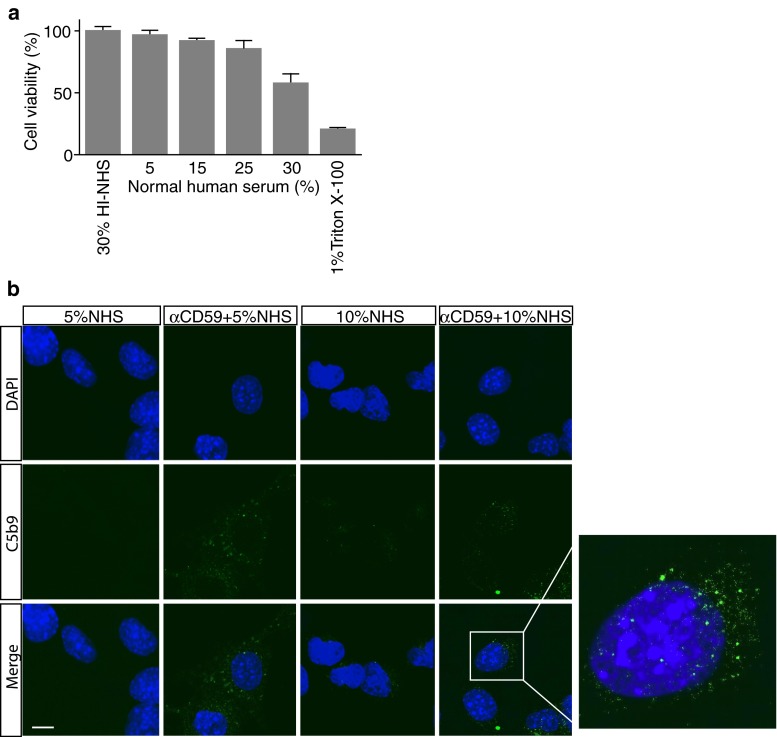
Formation of C5b-9 on 661W cells. **a** MTT viability assay measuring the extent of 661W cell death following treatment with blocking antibodies to CD59 and increasing concentrations (5, 15, 25, 30 %) of NHS for 1 h at 37 °C. For the negative control, cells were treated with anti-CD59 and 30 % HI-NHS, and for the positive control with 1 % Triton X-100. Data are plotted as mean ± S.D. (*n* = 3). **b** 661W cells were immunostained for C5b-9 and counterstained with DAPI to visualise nuclei. Cells were treated with 5 % NHS alone, anti-CD59+ 5 % NHS, 10 % NHS alone, or anti-CD59+ 10 % NHS. C5b-9 was barely visible at the lower serum concentration or in cells that weren’t treated with the CD59 blocking antibody, but appeared in punctate form in cells exposed to 10 % NHS (zoomed image). Scale bar = 10 μm. All experiments were repeated at least three times, and representative images are shown

To determine the tolerance of 661W cells to the terminal complement complex, cells were incubated first with rabbit polyclonal antiserum to CD59 and then switched to medium containing a range of concentrations of normal human serum (NHS), with 30 % heat-inactivated (HI)-NHS employed as control. The MTT assay shows that NHS was required to induce cell death (Fig. 2a), and that cell viability decreased with increasing concentrations of NHS, whereas treatment with the combination of anti-CD59 and HI-NHS had no effect on the cell viability. There was approximately 5 % cell death in the presence of 5 % NHS, and almost 50 % cell death in the presence of 30 % NHS. To confirm the presence of C5b-9 on the 661W cells under these experimental conditions we performed immunofluorescence analysis in conjunction with confocal microscopy, and observed characteristic punctate staining [15] only on 661W cells treated with the anti-CD59 blocking antibody and 5 or 10 % NHS, and not on cells treated with NHS alone (Fig. 2b).

### C5b-9 formation promotes apoptosis of 661W cells

**Fig. 3:**
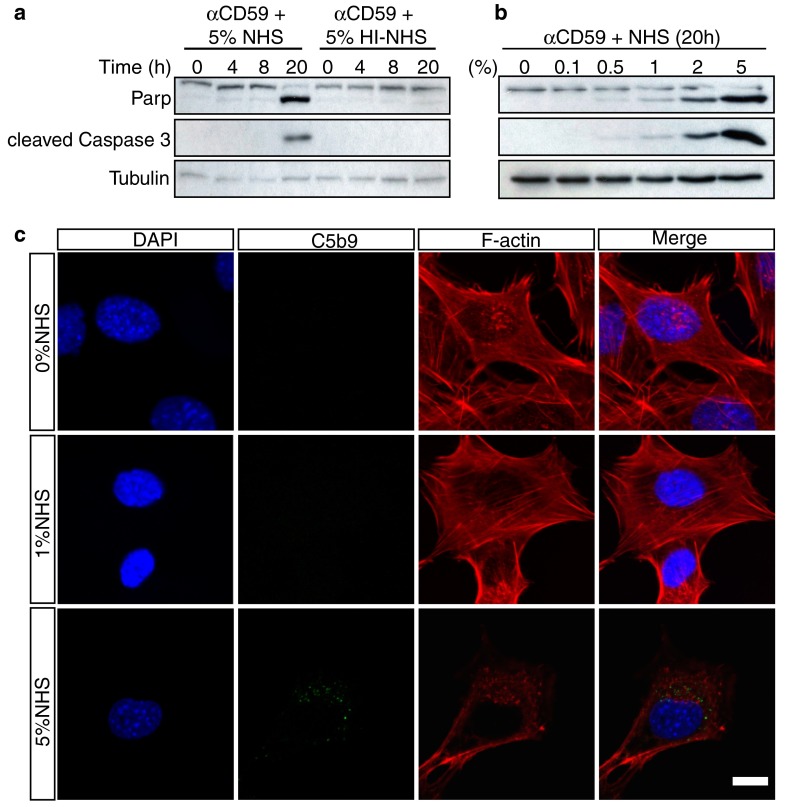
C5b-9 induces signature features of apoptosis in 661W cells in a dose- and time-dependent manner. **a** 661W cells were serum-starved for 24 h, then treated with anti-CD59 for 1 h followed by 5 % NHS for 1 h. Cells were lysed after 0, 4, 8 and 20 h and subjected to immunoblot analysis for PARP and cleaved caspase 3. Cells were treated with 5 % HI-NHS as a negative control and α-tubulin was included as a loading control. The blot shows that cleavage of PARP and activation of caspase-3 after 20 h occurred only in cells treated with NHS. **b** 661W cells were prepared as in (**a**), and treated with different concentrations of NHS for 20 h before immunoblotting. PARP cleavage and caspase-3 activation were faintly evident in 1 % NHS, but strikingly so in 2 and 5 % NHS. **c** 661W cells were prepared as in (**a**) with exposure to NHS for 20 h, then fixed, immunostained for C5b-9 and F-actin with nuclear counterstaining, and examined by confocal microscopy to investigate morphological changes. The images show that in 0 and 1 % NHS, 661W cells maintained a normal healthy morphology with abundant F-actin stress fibres, whereas in 5 % NHS surviving cells typically were smaller with fewer stress fibres and F-actin clumping. *Scale bar* 10 μm. All experiments were repeated at least three times, and representative blots/images are shown

To determine if C5b-9 formation promoted cell apoptosis, we used western blotting of whole cell lysates to examine poly (ADP-ribose) polymerase (PARP), a 116 kDa protein that is cleaved by activated caspase-3, and cleaved/activated caspase-3 as positive apoptotic markers. The blots showed that PARP and caspase-3 cleavage were evident 20 h after 1 h treatment of anti-CD59 and 5 % NHS respectively (Fig. 3a). In contrast, there was no PARP cleavage or caspase-3 activation when cells were treated with anti-CD59 and 5 % HI-NHS. These results show that C5b-9 formation is sufficient to stimulate 661W cell apoptosis, and are consistent with observations in animal models of retinal degeneration [16]. A dose–response experiment was also performed, and this showed that cleavage of PARP and activation of caspase-3 were just detectable in cells exposed to 0.5 % NHS and increased markedly up to 5 % NHS (Fig. 3b). We also investigated morphological changes in 661W cells under these conditions, and observed that 5 % NHS induced the formation of C5b-9, cell shrinkage and disassembly of F-actin stress fibres that was not observed in cells treated with 0 or 1 % NHS (Fig. 3c).

### C7 is required for C5b-9 formation and 661W apoptosis

**Fig. 4:**
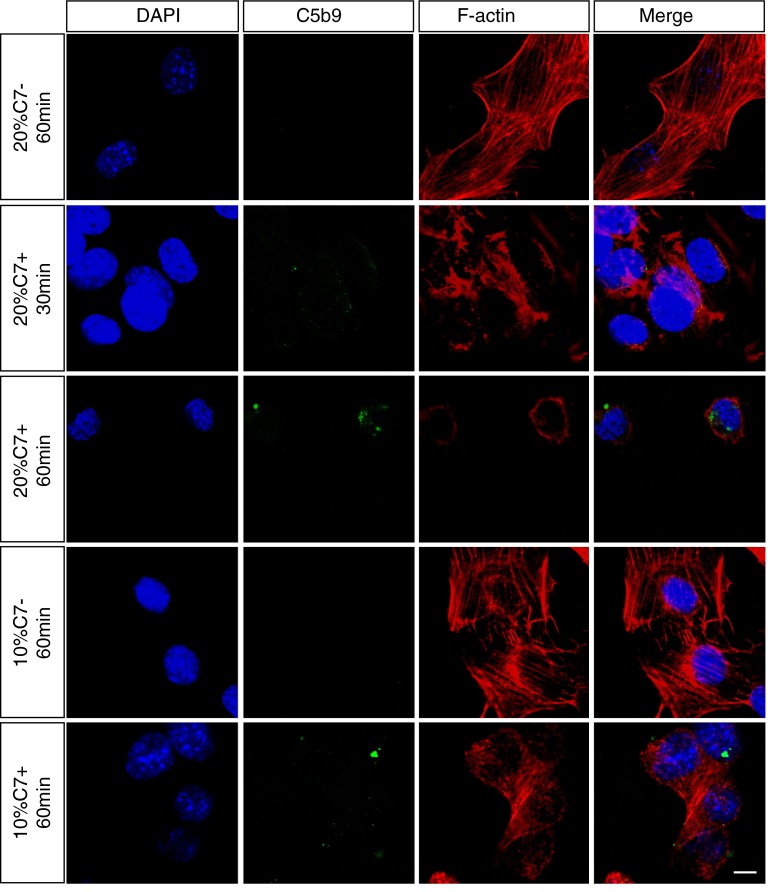
C7 is required for C5b-9 formation. Serum-deprived and anti-CD59 treated 661W cells were treated with either C7-depleted NHS (10 or 20 % for 60 min) or C7-depleted serum supplemented with purified exogenous C7 (100 µg/ml) (10 or 20 % for 60 min). Immunofluorescence staining and confocal imaging of C5b-9 and F-actin shows that in cells treated with C7 deficient serum, cellular morphology was normal with abundant F-actin stress fibres, and no evidence of C5b-9 staining. Restoration of C7 led to the appearance of punctate C5b-9 staining, and characteristic disruption of the F-actin cytoskeleton. DAPI was used to stain the nuclei. *Scale bar* 10 μm. All experiments were repeated at least three times, and representative images are shown

To confirm that 661W apoptosis was indeed induced by activation of complement, and not another heat-sensitive constituent of serum, we investigated C5b-9 assembly on 661W cells using C7-depleted normal human serum (C7-NHS) and C7-NHS supplemented with purified C7 (C7+NHS). Immunofluorescence analysis revealed no C5b-9 staining or morphological changes, as judged by F-actin staining, when cells were treated with 10 or 20 % C7-NHS (Fig. 4). In contrast, cells treated with either 10 or 20 % C7+NHS showed clear patchy C5b-9 staining and changes in morphology including shrinkage and clumping of F-actin bundles consistent with the early stages of apoptosis [17]. These experiments confirm that the effects of NHS that we observe are due to the presence of C5b-9 on 661W cells and not a non-specific labile component of serum.

### C5b-9 modulates 661W cell sensitivity to apoptosis

**Fig. 5:**
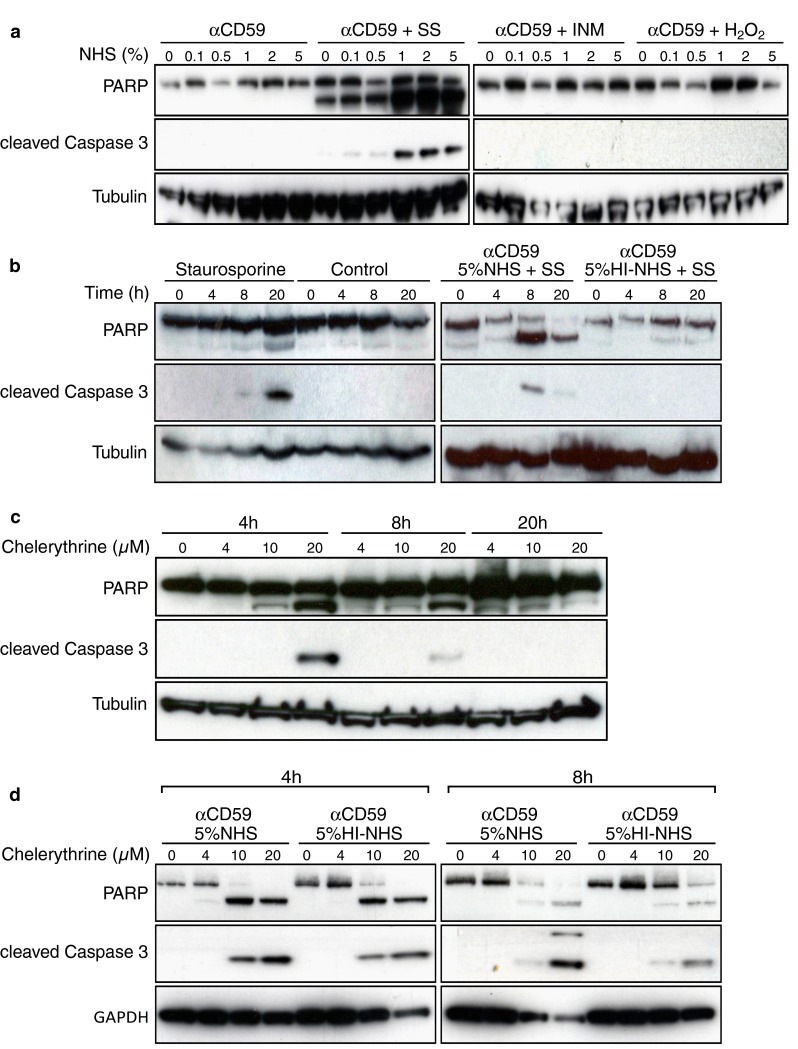
C5b-9 sensitizes 661W cells to staurosporine-mediated apoptosis. Cell apoptosis was detected by immunoblotting for PARP cleavage and activated caspase 3. **a** Serum-deprived and anti-CD59 treated 661W cells were incubated with increasing concentrations of NHS (0, 0.1, 0.5, 1, 2, 5 %) for 1 h, then with complete medium alone or with 100nM staurosporine (SS), 1 µM ionomycin (INM) or 10 µM H_2_O_2_ for 8 h. **b** 661W apoptosis was compared between cells treated with 100 nM staurosporine or medium alone for 0, 4, 8 and 20 h, which revealed PARP cleavage and caspase-3 activation only at 20 h. The kinetics of staurosporine-induced apoptosis were then investigated following treatment of 661W cells with either 5 % NHS or 5 % HI-NHS for 1 h, which revealed PARP cleavage and caspase-3 activation at 8 h only in the presence of 5 % NHS. **c** 661W cells were treated with a range of concentrations of chelerythrine for 4, 8 and 12 h. The immunoblots show rapid onset of PARP cleavage and caspase-3 activation with 10 and 20 µM agonist. **d** The sensitivity of 661W cells to chelerythrine was examined with and without prior exposure to 5 % NHS or 5 % HI-NHS. The immunoblots show that at 4 and 8 h, there was no difference between 5 % NHS and 5 % HI-NHS, though in both conditions PARP cleavage and caspase-3 activation were enhanced compared to chelerythrine alone (**c**)

Next we examined whether assembly of C5b-9 on the surface of 661W cells alters their sensitivity to agents known to induce apoptosis. As we had previously shown that there was no PARP cleavage or caspase-3 activation at 8 h post induction of C5b-9 formation in the presence of 5 % NHS (Fig. 3a), we therefore incubated 661W cells with various concentrations of NHS (0–5 %) for 8 h in the presence of staurosporine, ionomycin and H_2_O_2_. Figure 5a shows that of these three agonists only staurosporine induced PARP cleavage and caspase-3 activation, and that this was markedly enhanced in cells treated with higher concentrations of NHS (1, 2, 5 %).

We then asked whether PARP cleavage and caspase-3 activation were dependent on the combination of C5b-9 and staurosporine, or due solely to the staurosporine. Figure 5b shows that within the 8 h time frame staurosporine alone was insufficient to cause PARP cleavage and caspase-3 activation, but did cause modest PARP cleavage by 20 h. However, concomitant incubation with 5 % NHS greatly increased PARP cleavage and caspase-3 activation compared to 5 % HI-NHS. Several groups have reported that staurosporine can be used to induce 661W apoptosis [18, 19], with similar slow kinetics to those we observe here. Staurosporine is a broad-spectrum protein kinase inhibitor [20], but the mechanism whereby it induces apoptosis is not understood. We therefore compared the effects of staurosporine on 661W cells with chelerythrine, a more specific inhibitor of protein kinase C (PKC). We observed that chelerythrine alone induced PARP cleavage and caspase-3 activation in a dose- and time-dependent manner (Fig. 5c), but that in contrast to staurosporine, PARP cleavage and caspase-3 activation were not potentiated by C5b-9 (Fig. 5d).

Taken together these results reveal 661W cells to be remarkably resistant to levels of calcium cytotoxicity and oxidative stress that would readily kill many other cell types, but to be more susceptible to protein kinase inhibitors, with prior formation of C5b-9 sensitizing 661W cells to staurosporine but not to chelerythrine.

### 661W cells die by both apoptosis and necroptosis

**Fig. 6:**
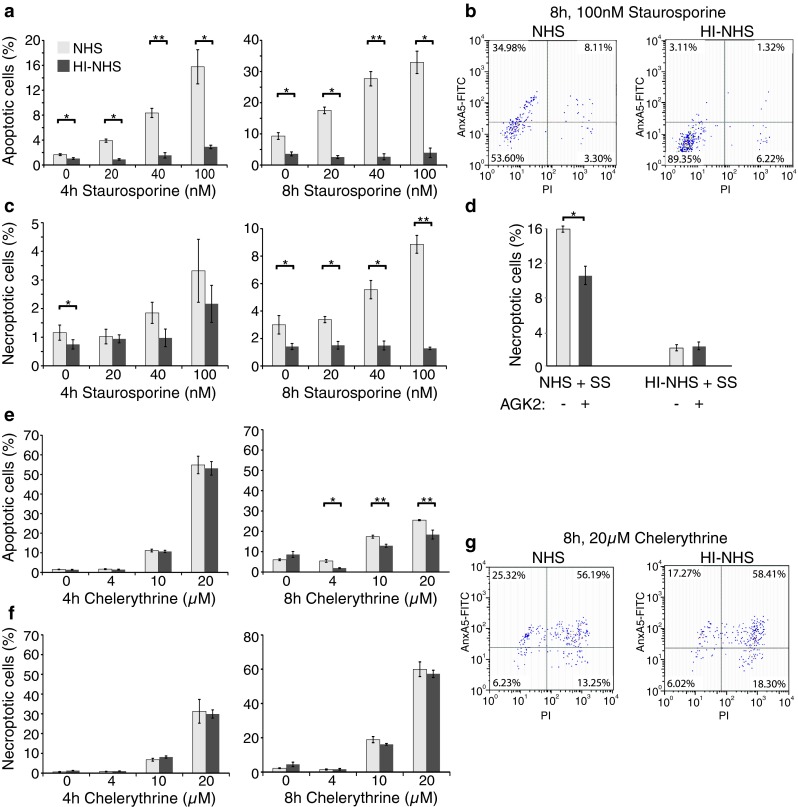
Chelerythrine and staurosporine induce apoptosis and necroptosis in 661W cells. Cell death was examined by Annexin V/PI staining and flow cytometry. **a** Quantitation of apoptotic 661W cells following treatment with a range of concentrations of staurosporine (0–100 nM) for 4 h and 8 h in the presence of 5 % NHS or 5 % HI-NHS. **b** Example of the primary data for the 8 h, 100 nM staurosporine experiment. Apoptotic cells are Annexin V^+^ and locate to the top-left quadrant of the scatter plot whereas necroptotic cells are Annexin V and PI double positive and locate to the upper right quadrant. **c** Quantitation of necroptotic 661W cells from the same experiment as (**a**) and (**b**). **d** Necroptotic 661W cells were quantified as in (**c**) following treatment with 100 nM staurosporine for 8 h ± 10 µM AGK2. Necroptosis was significantly inhibited by AGK2. **e** Serum-starved and anti-CD59 treated 661W cells were incubated with 5 % NHS or 5 % HI-NHS for 1 h and then incubated with 0–20 µM chelerythrine for 4 or 8 h. Cells were then stained and subjected to flow cytometry as described above. The bar charts show the percentages of apoptotic cells. **f** Quantitation of necroptotic 661W cells from the same experiment as (**e**). **g** Representative scatter plots showing the results for cells treated with 20 µM chelerythrine for 8 h. (*P < 0.05, **P < 0.01, *n* = 3)

To more accurately quantify 661W death and to corroborate the results of the western blots in Fig. 5, we used flow cytometry with annexin V (AnxV) and propidium iodide (PI) staining to identify cells undergoing apoptosis and necrosis respectively. Figures 6a, b confirmed that 5 % NHS, but not 5 % HI-NHS, significantly increased the percentage of apoptotic cells (AnxV^+^ PI^−^) (*P < 0.05, **P < 0.01) in the presence of staurosporine (0, 20, 40 and 100 nM) at both 4 and 8 h. Notably, the fold differences in the number of apoptotic cells when comparing NHS and HI-NHS were approximately 4–5 (4 h) and 6–8 (8 h) in the presence of staurosporine, but only 1–2 (4 h) and 2–3 (8 h) in the absence of staurosporine. Intriguingly, we observed many fewer cells positive for PI alone, but a significant pool of cells (~8 %) that were AnxV^+^ PI^+^, a hallmark characteristic of necroptosis, or programmed necrosis (Fig. 6c). The necroptotic and apoptotic pools were evident only in the presence of C5b-9, and generation of the necroptotic pool was significantly inhibited by AGK2 (*P < 0.05, Fig. 5d), a pharmacological inhibitor of SIRT2 deacetylase activity [21] that has recently been shown to be required for programmed necrosis [22]. In contrast to the results obtained with staurosporine, the sensitivity of 661W cells to the PKC inhibitor chelerythrine was much less affected by the presence of C5b-9. Thus, chelerythrine rapidly induced both apoptosis and necroptosis, with the number of apoptotic (AnxV^+^ PI^−^) cells exceeding necroptotic (AnxV^+^ PI^+^) cells at 4 h, with the converse at 8 h (Figs. 6e–g). A small but significant increase in the number of apoptotic cells was associated with C5b-9 after 8 h treatment with chelerythrine (**P < 0.01, Fig. 6e).

## Discussion

Photoreceptor cell death is responsible for the majority of registered blindness and vision loss, so there is a need to improve our understanding of the cellular mechanisms that drive this process in order to develop new and more effective therapies. Recently it has become clear that although apoptosis may account for a significant fraction of photoreceptor loss, other pathways, in particular necrosis and necroptosis, appear to play a significant role [23]. Photoreceptor loss in AMD is generally considered to occur secondary to retinal pigment epithelial (RPE) cell death or dysfunction caused by inadequate control of complement activation [13]. There is therefore a clinical parallelism in AMD pathology in which RPE cells and cone photoreceptors both degenerate, though not necessarily simultaneously. Whilst little is known about the effects of complement activation on photoreceptors, studies on C5b-9 formation on RPE cells have reported both cell death at high levels, and modulation of RPE gene expression at lower, sublytic levels [24, 25]. Whilst the RPE/Bruch’s membrane may be the primary site of complement-mediated cell damage, the expression of complement regulatory proteins in the neural retina [6] suggests that in disease, photoreceptors may themselves become vulnerable to direct assault from C3 and its breakdown products or C5b-9.

In this study we describe a protocol for the assembly of sub-lytic levels of C5b-9 on 661W cells. C5b-9, or membrane attack complex, forms pores in cell membranes but most nucleated cells have mechanisms to escape lysis induced by C5b-9, typically eliminating the cell surface complex either by vesicular shedding or endocytosis and lysosomal degradation [15, 26]. However, we found that 661W cells underwent limited apoptosis as a consequence of C5b-9 exposure in the absence of any other insult, albeit relatively slowly. We also observed that C5b-9 rendered 661W cells particularly sensitive to death by staurosporine, a broad-specificity protein kinase inhibitor that has been shown to induce apoptosis in many cell types, including 661W cells [18, 19]. Here, we also observed apoptosis of 661W cells, as judged by PARP cleavage, activation of caspase-3 and AnxV positivity in C5b-9 treated cells exposed to staurosporine.

Although we found 661W cells to be highly resistant to oxidative stress, flow cytometric analysis revealed that both staurosporine and chelerythrine induced not only AnxV^+^ cells typically associated with apoptosis, but also AnxV^+^ PI^+^ cells. Positivity for PI alone is a characteristic of necrosis, but the double positivity observed here is consistent with programmed necrosis, or necroptosis, suggesting that staurosporine and chelerythrine can simultaneously induce both apoptosis and necroptosis in 661W cells. Interestingly, 661W cells have also been reported to undergo cell death by necrosis in response to light exposure [27, 28]. Necroptosis is a form of cell death particularly associated with neurodegenerative disease that may be initiated by death receptors such as the tumour necrosis factor receptor 1 in association with the RIP1 and RIP3 kinases [29, 30]. The NAD-dependent deacetylase SIRT2 has also recently been shown to be required for necroptosis [22], and here we showed that the SIRT2 inhibitor AGK2 attenuated the generation of AnxV^+^ PI^+^ 661W cells in response to staurosporine. Our observation that staurosporine can induce both apoptosis and necroptosis in complement-sensitized 661W cells is consistent with reports that both forms of cell death have been observed in response to staurosporine in rat astrocytes [31], and also in our own prior in vivo observations of AnxV^+^ PI^+^ RGCs following intravitreal injection of either staurosporine or amyloid ß in mouse eyes [32].

## Conclusions

In conclusion, the results of our study add weight to the idea that complement dysregulation may play a role in the molecular pathogenesis of retinal diseases characterised by the death of cone photoreceptors. In AMD, where complement is already clearly implicated in a causative role, loss of macular cones is a major cause of vision loss and our results here suggest that complement activation may either directly cause cone cell death, or sensitize cones to other insults. Our results also suggest that apoptosis may not be solely responsible for cone cell death in retinal degeneration. Although we identified apoptosis and necroptosis using staurosporine, which is not a physiological agonist, necroptosis is strongly linked to tumour necrosis factor-α (TNF-α) signalling [33, 34], and aberrant TNF-α signalling has been extensively linked to retinal degeneration [27, 35, 36] and AMD [37]. In future work we will use rodent models of cone loss to investigate whether there is a functional link between complement dysregulation, TNF-α signalling and necroptosis.
